# The complete chloroplast genome of *Camellia leyeensis* (theaceae)

**DOI:** 10.1080/23802359.2022.2068980

**Published:** 2022-05-02

**Authors:** Xu Xiao, Jiangtao Lu, Guoyuan Yang, Zhi Li

**Affiliations:** a College of Forestry, Guizhou University, Guiyang, Guizhou Province, China; b Biodiversity and Nature Conservation Research Center, Guizhou University, Guiyang, Guizhou Province, China

**Keywords:** *Camellia leyeensis*, chloroplast genome, phylogenetic analysis

## Abstract

*Camellia leyeensis* Chang ＆ Y. C. Zhong is a plant belonging to the genus *Camellia.* To determine its correct taxonomic status and better understand its molecular phylogenetic and genetic diversity, we studied the chloroplast genome of this species. Here, we report and characterize the complete chloroplast (cp) genome of *C. leyeensis* by using Illumina paired-end sequencing data. The chloroplast genome was determined to be 157,063 bp in length with a GC content of 37.30%. The genome contained 136 genes, including 91 protein-coding genes, 37 tRNA genes, and 8 rRNA genes. The sequence contained a large single-copy region (LSC, 86,661 bp), a small single-copy region (SSC, 18,284 bp), and two inverted repeat sequences (IRs, 26,059 bp, each). The GC content of the IR regions (42.96%) was higher than that of the SSC region (30.6%) and LSC region (35.31%). Phylogenetic analysis suggested that *Camellia leyeensis* is closely related to *C. anlungensis* with 96% bootstrap support. This chloroplast genome would be helpful for the phylogeny and conservation of *Camellia*.

The genus *Camellia* of the family Theaceae comprises approximately 120 species, most of which have high economic and ornamental value (e.g. *Camellia. sinensis* (L.) O. Ktze. and *Camellia oleifera* Abel.). These species are divided into 20 sections based on morphological characteristics and genetic relationships. Approximately 97 species of *Camellia* are distributed in southern and southwestern China (76 endemic) (Ming and Bartholomew 2007). *Camellia leyeensis* Chang et Y. C. Zhong was first reported as a new species in 1991 belonging to Sect. *Tuberculata*, *Camellia* genus. It is different from species in other sections of *Camellia* in terms of its morphological features, specifically, having tuberculate capsules. This species, which commonly grows atop mountains with evergreen broad-leaved forests, is mainly distributed in the Ya-an Forest Farm, Leye County, Guangxi, with *Pinus massoniana* Lamb. and *Celtis sinensis* Pers and other shrubs as companion species. This taxon is similar to *Camellia rhytidocarpa* Chang et Liang. However, it can be distinguished from *C. rhytidocarpa* by its elliptical leaves and small capsules. Current studies on *C. leyeensis* have focused only on its leaf anatomy and micromorphological characteristics and biogeography (Lu et al. 2008). However, there are few reports of other studies on *C. leyeensis*. In this study, for the first time, we reported the chloroplast genome sequence of *C. leyeensis* provided useful genomic resources not only for the exchange of information between different species, but also for its phylogenetic and evolutionary studies.

Total DNA was isolated from fresh leaves of a *C. leyeensis* individual collected from its type locality, which is Ya-an Forest Farm, Leye County, Guangxi Province (24.85.833 N, 106.29.208 E, alt. 684 m). The specimen and extracted DNA were deposited at the Biodiversity Research Laboratory, Forestry College of Guizhou University (http://fc.gzu.edu.cn) under voucher number LZ20210701 (collected by Zhi Li and Xu Xiao, lizhighz@163.com and 1758257279@qq.com). The guidelines established by the Administration of Affairs Concerning Plant Experimentation state that approval from the Science and Technology Bureau of China and the Department of Wildlife Administration is not necessary when the plant in question are neither rare nor near extinction (first- or second-class state protection level). Therefore, approval was not required for the experiments described in this paper. Total genomic DNA was extracted from fresh leaves by using the modified CTAB procedure of Doyle and Doyle (1987) and sequenced on the Illumina NovaSeq 6000 platform (Illumina, San Diego, CA). Genome sequences were screened and assembled with SPAdes v.3.5.0 (Lapidus A. et al. 2014).

The complete cp genome of *C. leyeensis* (GenBank accession OK046127) is 157,063 bp in size, with a GC content of 37.30%, similar to the chloroplast genome of most *Camellia* genus plants (Li et al. 2018; Yayan et al. 2020). The annotated complete cp genome contains 136 genes, including 91 protein-coding genes (PCGs), 37 transfer RNAs (tRNAs), and 8 ribosomal RNAs (rRNAs), accounting for 66.91%, 27.21%, and 5.88% of all annotated genes, respectively. The sequence is composed of a large single-copy region (LSC, 86,661 bp), a small single-copy region (SSC, 18,284 bp), and two inverted repeat sequences (IRs, 26,059 bp, each). The GC content of the IR regions (42.96%) is higher than that of the SSC region (30.6%) and LSC region (35.31%).

To perform a phylogenomic analysis, 29 Theaceae family species plastomes were obtained from GenBank for maximum-likelihood (ML) phylogenetic analysis, and *Polyspora hainanensis* (Hung T. Chang) C. X. Ye ex B. M. Bartholomew & T. L. Ming, was used as an outgroup. The alignment was performed using MAFFT (Katoh and Standley 2013). The phylogenetic tree was built using RAxML8.1.5 (Stamatakis 2014) with bootstrap values set to 1000. The results showed that *C. leyeensis* is sister to *C. anlungensis* with 96% bootstrap support (Figure 1).

**Figure 1.**
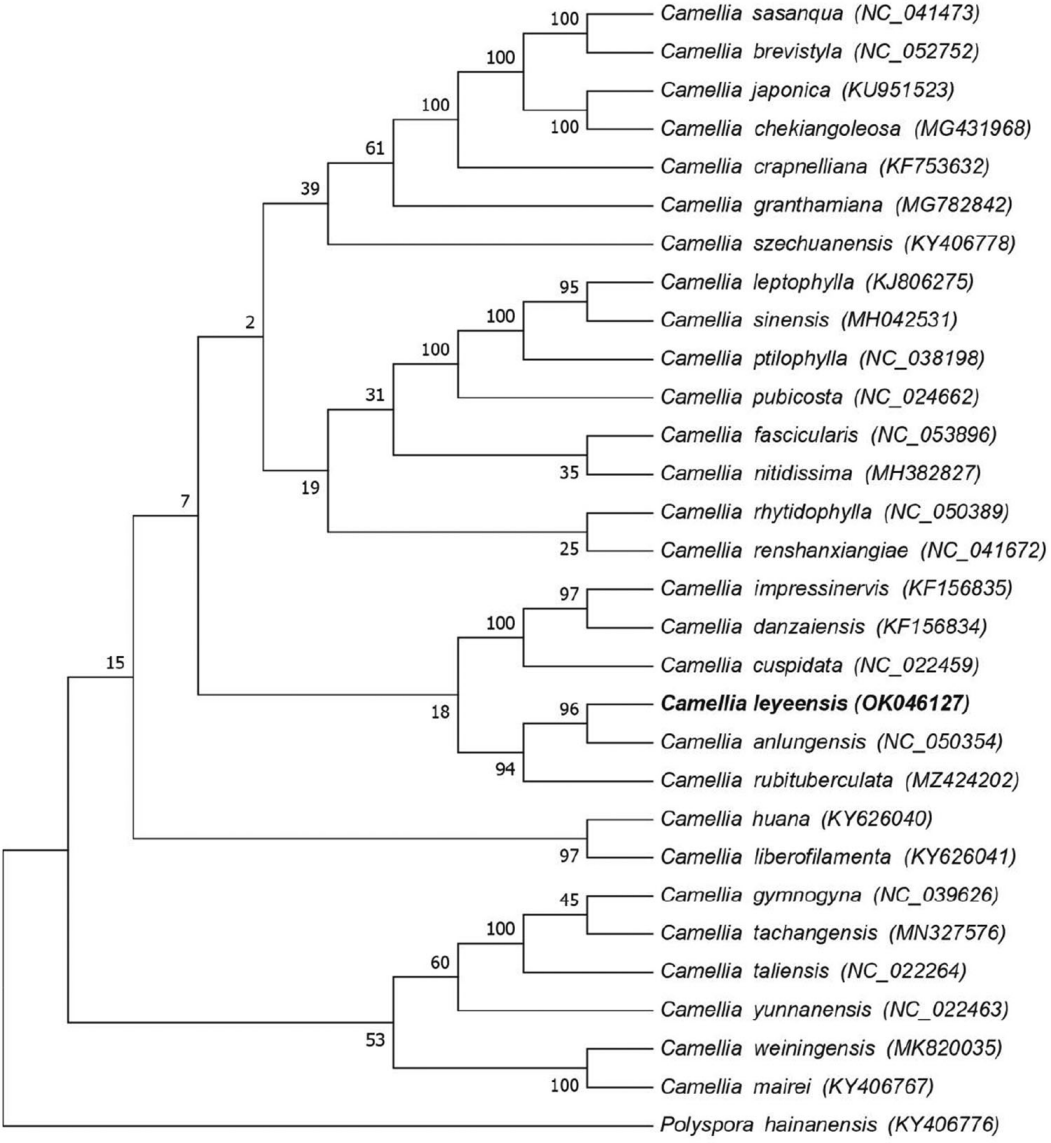
Maximum likelihood tree of *C. leyeensis* based on whole chloroplast genome sequences; *Polyspora hainanensis* is the outgroup. Numbers near the nodes represent ML bootstrap values.

## Author contributions

Field work, Conceived and designed the experiments: Zhi Li and Xu Xiao; Performed the experiments: Jiangtao Lu and Guoyuan Yang; Analyzed the data and wrote the manuscript: Xu Xiao and Zhi Li.

## Data Availability

The data used to support the findings of this study are available from the corresponding author upon request and in GenBank at [https://www.ncbi.nlm.nih.gov/genbank/] (accession number OK046127). The associated BioProject, SRA, and Bio-Sample numbers are PRJNA765056, SRR16037759, and SAMN21543270, respectively.
